# Fatty Acid-binding Proteins Interact with Comparative Gene Identification-58 Linking Lipolysis with Lipid Ligand Shuttling*

**DOI:** 10.1074/jbc.M114.628958

**Published:** 2015-05-07

**Authors:** Peter Hofer, Andras Boeszoermenyi, Doris Jaeger, Ursula Feiler, Haribabu Arthanari, Nicole Mayer, Fabian Zehender, Gerald Rechberger, Monika Oberer, Robert Zimmermann, Achim Lass, Guenter Haemmerle, Rolf Breinbauer, Rudolf Zechner, Karina Preiss-Landl

**Affiliations:** From the ‡ Institute of Molecular Biosciences, University of Graz, NAWI Graz, 8010 Graz, Austria,; the § Department of Biological Chemistry and Molecular Pharmacology, Harvard Medical School, Boston, Massachusetts 02115,; the ¶ Institute of Organic Chemistry, Graz University of Technology, 8010 Graz, Austria, and; the ‖ NanoTemper Technologies GmbH, 81369 Munich, Germany

**Keywords:** adipose triglyceride lipase (Atgl), fatty acid-binding protein, lipid signaling, lipolysis, peroxisome proliferator-activated receptor (Ppar)

## Abstract

The coordinated breakdown of intracellular triglyceride (TG) stores requires the exquisitely regulated interaction of lipolytic enzymes with regulatory, accessory, and scaffolding proteins. Together they form a dynamic multiprotein network designated as the “lipolysome.” Adipose triglyceride lipase (Atgl) catalyzes the initiating step of TG hydrolysis and requires comparative gene identification-58 (Cgi-58) as a potent activator of enzyme activity. Here, we identify adipocyte-type fatty acid-binding protein (A-Fabp) and other members of the fatty acid-binding protein (Fabp) family as interaction partners of Cgi-58. Co-immunoprecipitation, microscale thermophoresis, and solid phase assays proved direct protein/protein interaction between A-Fabp and Cgi-58. Using nuclear magnetic resonance titration experiments and site-directed mutagenesis, we located a potential contact region on A-Fabp. In functional terms, A-Fabp stimulates Atgl-catalyzed TG hydrolysis in a Cgi-58-dependent manner. Additionally, transcriptional transactivation assays with a luciferase reporter system revealed that Fabps enhance the ability of Atgl/Cgi-58-mediated lipolysis to induce the activity of peroxisome proliferator-activated receptors. Our studies identify Fabps as crucial structural and functional components of the lipolysome.

## Teaser

**Background:** A multiprotein complex designated as lipolysome degrades intracellular triglycerides and contains proteins such as adipose triglyceride lipase (Atgl) and its co-activator Cgi-58.

**Results:** Cgi-58 interacts with fatty acid-binding proteins (Fabps), which impact Atgl-mediated lipolysis and lipid signaling.

**Conclusion:** Fabps modulate Atgl-mediated TG hydrolysis and link lipolysis with intracellular lipid ligand shuttling.

**Significance:** Novel mechanistic insights into the regulation of lipid catabolism and energy homeostasis are presented.

## Introduction

Intracellular lipolysis includes the stepwise hydrolytic breakdown of triglycerides (TGs),^3^ stored in lipid droplets (LDs), resulting in the formation of glycerol and fatty acids (FAs). TG catabolism involves the consecutive action of adipose triglyceride lipase (Atgl), which hydrolyzes TGs to diglycerides, hormone-sensitive lipase (Hsl) converting diglycerides to monoglycerides, and monoglyceride lipase, hydrolyzing monoglycerides to FA and glycerol (1). These enzymes constitute the core of a multiprotein network (1, 2) encompassing a variety of regulatory, accessory, and scaffolding proteins (3–5). Lipolysis basically occurs in all cell types and tissues to meet the cellular requirements for FAs as energy substrates or building blocks for membrane lipids. It is most prominent in white adipose tissue (WAT) that supplies oxidative tissues with FAs via the bloodstream during times of starvation and physical exercise. Lipolysis is a tightly regulated process, and it critically depends on specific protein/protein interactions. Such transient complexes are common in the regulation of metabolic pathways and signaling cascades enabling instant cellular responses to diverse stimuli (6, 7). Additionally, metabolic lipases hydrolyzing TGs often require specific co-activators for maximal enzyme activity, presumably by mediating enzyme/substrate interaction at the water-lipid interphase. Examples include the activation of pancreatic lipase by colipase or lipoprotein lipase by apolipoprotein CII (8, 9).

For full stimulation of intracellular lipolysis, the initiating enzyme Atgl needs comparative gene identification-58 (Cgi-58) as co-activator. This 39-kDa protein directly binds to Atgl and strongly stimulates its hydrolytic activity by a currently unknown mechanism (10–12). Atgl stimulation during β-adrenergic- and exercise-induced induction of lipolysis occurs via its interaction with Cgi-58 on LDs (13, 14), following Cgi-58 detachment from perilipin-1 (15, 16). The lipolysome also contains an inhibitory protein blocking Atgl activity, named the G0/G1 switch gene 2 (G0s2). This 103-amino acid protein was originally identified as a cell cycle progression factor but was later found to bind directly to Atgl, independently of the presence of Cgi-58 (17–20). Other documented Atgl interaction partners include UBX domain-containing protein 8 (Ubxd8) (4), pigment epithelium-derived factor (Pedf) (5, 21, 22), and the Golgi brefeldin A resistance factor-1 (Gbf-1) (23), supporting the concept that functional lipolysis requires a complex cooperation of a multifactorial protein network.

Here, we demonstrate by multiple experimental approaches that Cgi-58 interacts with adipocyte-type (A-Fabp) and other fatty acid-binding proteins. The physiological consequences of this interaction include the stimulation of lipolysis and the activation of peroxisome proliferator-activated receptor (Ppar)-mediated gene transcription.

## Experimental Procedures

### 

#### 

##### Label Transfer Experiments

To obtain tissue lysates, gonadal WAT samples were washed in 1× PBS, homogenized on ice in solution A (0.25 m sucrose, 1 mm EDTA, 20 μg/ml leupeptin, 2 μg/ml antipain, 1 μg/ml pepstatin, pH 7.0) using Ultra Turrax (IKA, Staufen, Germany), and centrifuged for 30 min at 20,000 × *g* at 4 °C. To prepare the bait protein for the label transfer, purified mouse Gst-Cgi-58 (13) was covalently linked to the hetero-bifunctional cross-linker Sulfo-SBED following the manufacturer's instructions (Sulfo-SBED biotin label transfer reagent kit, Thermo Scientific, Waltham, MA). The modified bait protein was incubated with the tissue lysate, photoactivation performed at 312 nm for 5 min, and the DTT concentration adjusted to 50 mm to allow transfer of the biotin tag. The biotinylated proteins were enriched using streptavidin-agarose (Invitrogen), separated using SDS-PAGE, and digested in gel with trypsin (24). The resulting peptides were analyzed by LC-MS/MS as described by Birner-Gruenberger *et al.* (25). Data analysis was performed by using the Spectrum Mill software (Agilent, Waldbronn, Germany). The database used was the “mouse” subset of “SwissProt” database from ncbi.nih.gov.

##### Cloning of Recombinant fabps

The coding sequences for human *l-, i-, h-, a*-, and *e-fabps* were amplified by PCR from human cDNA using PHUSION-Polymerase (Biozym Scientific, Oldendorf, Germany). Primers were designed to contain endonuclease cleavage sites for subsequent cloning into the bacterial Strep-tag expression vector pASK-IBA5plus (IBA GmbH, Göttingen, Germany) as follows: *l-fabp* forward 5′-GACGACGGTACCAAGTTTCTCCGGCAAGTACCA-3′ and *l-fabp* reverse 5′-GACGACGGATCCTTAAATTCTCTTGCTGATTCTCTTG-3′, *i-fabp* forward 5′-GCGACGAATTCAGCGTTTGACAGCACTTGGAA-3′ and *i-fabp* reverse 5′-GACGACGGATCCTCAATCCTTTTTAAAGATCCTTTTG-3′;*h-fabp* forward 5′-GACGACGAATTCAGTGGACGCTTTCCTGGGCAC-3′ and *h-fabp* reverse 5′-GACGACGGATCCTCATGCCTCTTTCTCATAAGTGCGA-3′; *a-fabp* forward 5′-GACGACGAATTCATGTGATGCTTTTGTAGGTAC-3′ and *a-fabp* reverse 5′-GACGACGGATCCTTATGCTCTCTCATAAACTC-3′; *e-fabp* forward 5′-GACGACGAATTCAGCCACAGTTCAGCAGCTGGA-3′ and *e-fabp* reverse 5′-GACGACGGATCCTTATTCTACTTTTTCATAGATCCGAGTACA-3′.

##### Site-directed Mutagenesis

All mutant variants were generated using the GENEART site-directed mutagenesis system (Invitrogen) according to the manufacturer's instructions. The following primers were designed to introduce the point mutations: A-Fabp^R127Q^ forward 5′-TGAAAGGCGTCACTTCCACGCAAGTTTATGAGA-3′ and A-Fabp^R127Q^ reverse 5′-CGTGGAAGTGACGCCTTTCATGACGCATTC-3′; A-Fabp^F58A^forward 5′ TGAAAGTACCGCCAAAAATACTGAGATTTCC-3′ and A-Fabp^F58A^ reverse 5′-GATTTAATGGTGATCACATCC-3′; A-Fabp^K22E^ forward 5′-AACTTTGATGATTATATGGAAGAAGTAGGAGTGGGCTTT-3′ and A-Fabp^K22E^ reverse 5′-AAAGCCCACTCCTACTTCTTCCATATAATCATCAAAGTT-3′; A-Fabp^R31E^ forward 5′-GGAGTGGGCTTTGCCACCGAAAAAGTGGCTGGCATGGCC-3′ and A-Fabp^R31E^ reverse 5′-GGCCATGCCAGCCACTTTTTCGGTGGCAAAGCCCACTCC-3′; A-Fabp^K22E,R31E^ forward 5′-GATTATATGGAAGAAGTAGGAGTGGGCTTTGCCACCGAAAAAGTGGCT-3′ and A-Fabp^K22E,R31E^ reverse; and 5′-AGCCACTTTTTCGGTGGCAAAGCCCACTCCTACTTCTTCCATATAATC-3′ and A-Fabp^D18K^ forward 5′-CCAGTGAAAACTTTGATAAGTATATGAAAGAAAGAAGTAGGAGTGGGC-3′, A-Fabp^D18K^ reverse 5′-GCCCACTCCTACTTCTTTCATATACTTATCAAAGTTTTCACTGG-3′. The numbering of the amino acids started with methionine as the first amino acid. Site-directed mutagenesis resulted in single amino acid exchanges except for A-Fabp^K22E,R31E^ with two amino acids changes.

##### Cloning of the cgi-58_S239E Variant with Increased Solubility

Mouse *cgi-58* (m*cgi-58*, coding for Lys-2–Asp-351 (UniProt ID Q9DBL9)) (13), preceded by a TEV protease cleavage site, was amplified by PCR and the primers forward 5′-CGAAGCAGAGAGCTCGAAAACCTGTATTTTCAGG-3′ and reverse 5′-GGAACCCTCGAGTCATCAGTCTACTGTGTGGC-3′. The TEV-m*cgi-58*-containing PCR product and a His-pSumo (kindly provided by C.D. Lima, Sloan-Kettering Institute) vector lacking a functional *Bam*HI endonuclease cleavage site were digested with the appropriate endonucleases and ligated with T4 ligase (New England Biolabs, Ipswich, MA) to yield His-pSumo-TEV-m*cgi-58*. The mutation S239E was introduced using the QuikChange® site-directed mutagenesis kit (Stratagene, La Jolla, CA) with the forward primer 5′-CCTGATTTCAAGCGGAAGTACGAGTCTATGTTTGAAGATGACACG-3′ and the reverse primer 5′-CGTGTCATCTTCAAACATAGACTCGTACTTCCGCTTGAAATCAGG-3′. The fusion protein encoded by the resulting construct His-pSumo-TEV-*cgi-58_S239E* was subsequently cleaved by the TEV protease to obtain the mutant variant mCgi-58^S239E^, which showed enhanced solubility, and was used for NMR experiments only. For reasons of simplification, this variant is called m^sol^Cgi-58 throughout the text.

##### Expression and Labeling of m^sol^Cgi-58 and A-Fabp

m^sol^Cgi-58 was co-expressed with the chaperone Tig encoded by pTf16 (Takara Bio Inc., Seta, Japan) in *Escherichia coli* BL21(DE3) cells at 37 °C in the presence of kanamycin (50 μg/ml), chloramphenicol (34 μg/ml), and l-arabinose (1 g/liter), to induce Tig expression. At *A*_600_ = 0.6, m^sol^Cgi-58 expression was induced with 0.5 mm isopropyl 1-thio-β-d-galactopyranoside for 9–12 h at 30 °C.

A-Fabp for NMR experiments was expressed in *E. coli* BL21(DE3) at 37 °C in M9 medium containing 50 μg/ml ampicillin, 1 g/liter, ^15^NH_4_Cl and 2 g/liter d-[^13^C]glucose (Cambridge Isotope Laboratories, Tewksbury, MA). Expression of the ^15^N- and ^13^C labeled A-Fabp was induced with 200 ng/ml anhydrotetracycline at an *A*_600_ = 0.6 and for 3 h.

##### Purification of m^sol^Cgi-58 for NMR Titration

Cells expressing m^sol^Cgi-58 were disrupted by sonication in wash buffer (20 mm Tris-HCl, 500 mm NaCl, 30 mm imidazole, 0.1% Nonidet P-40, 3.5 mm β-mercaptoethanol, pH 7.8), supplemented with protease inhibitor mixture (Complete, EDTA-free Tabs-Roche, Roche Diagnostics, Basel, Switzerland), 750 units benzonase® nuclease HC (Merck, Darmstadt, Germany), and 1 mg/ml lysozyme. After centrifugation at 30,000 × *g* for 40 min, m^sol^Cgi-58 was purified by affinity chromatography using the His-Trap FF column (GE Healthcare). The recombinant protein was eluted with a gradient of elution buffer (20 mm Tris-HCl, 500 mm NaCl, 250 mm imidazole, 10% glycerol, 3.5 mm β-mercaptoethanol, pH 7.8). After TEV cleavage (4 h at room temperature) m^sol^Cgi-58 was further purified by gel filtration Superdex 200 (Sigma), in gel filtration buffer (15 mm Na_2_HPO_4_/KH_2_PO_4_, pH 7.8, 300 mm NaCl, 1 mm DTT, 1 mm EDTA). m^sol^Cgi-58 samples were concentrated, and the buffer was exchanged to NMR titration buffer (15 mm Na_2_HPO_4_/KH_2_PO_4_, pH 7.0, 300 mm NaCl, 1 mm DTT, 1 mm EDTA).

##### Expression and Purification of Gst-Cgi-58 in E. coli

Gst-tagged Cgi-58 and truncation variants thereof used in solid phase assays were expressed and purified as described previously (13).

##### Purification of Strep-tagged Fabps

Plasmid-containing *E. coli* BL21(DE3) cells were disrupted by sonication in buffer W (100 mm Tris, pH 8.0, 150 mm NaCl, 1 mm EDTA, 20 μg/ml leupeptin, 2 μg/ml antipain, 1 μg/ml pepstatin). Soluble Strep-tagged proteins were purified by affinity chromatography using Strep-Tactin Superflow high capacity resin (IBA, Göttingen, Germany). Bound proteins were eluted in buffer E (100 mm Tris-HCl, pH 8.0, 150 mm NaCl, 10 mm desthiobiotin, 1 mm EDTA, 20 μg/ml leupeptin, 2 μg/ml antipain, 1 μg/ml pepstatin).

##### Expression of Recombinant Proteins in Eukaryotic Cells and Preparation of Cell Extracts

Monkey embryonic kidney cells (COS-7, ATCC CRL-1651) were cultivated in Dulbecco's modified Eagle's medium (DMEM) (Invitrogen) supplemented with 10% FCS (Sigma), penicillin (100 IU/ml), and streptomycin (100 μg/ml) in a standard humidified 5% CO_2_ atmosphere at 37 °C. For transfection, 6 μg of plasmid DNA were complexed to 30 μl of Metafectene (Biontex GmbH, Munich, Germany), unless otherwise stated, and the mixture was added to serum-free cell culture medium. After 4 h, medium was replaced with standard medium containing 10% FCS. Cells were harvested after 24–48 h, suspended in solution A, disrupted by sonication (Missonix sonicator, QSonica LLC, Newtown, CT), and centrifuged at 13,000 × *g* for 15 min. Protein concentrations were determined by protein assay (Bio-Rad, Munich, Germany) according to the manufacturer's protocol using BSA as standard.

##### NMR Resonance Assignment

Backbone resonance assignment and interaction mapping by NMR were performed with 500 μm ^15^N- and ^13^C-labeled A-Fabp samples in buffer 4 (100 mm NaPP, 150 mm NaCl, pH 6.5) and NMR titration buffer, respectively. 10% D_2_O was added to all samples. Spectra were recorded on a Varian/Agilent Inova 600 MHz spectrometer equipped with a cryogenically cooled probe and a 500 MHz Bruker spectrometer equipped with a TXI room temperature probe at 298 K, respectively. Backbone assignment was obtained by analysis of three-dimensional NMR experiments (HNCA/HNCOCA, HNCO/HNCACO, HNCACB, and CCONH experiments (26)). Backbone experiments were recorded nonuniformly with 15% Poisson gap weighted sampling of the grid in indirect dimensions (27) and reconstructed with the iterative soft thresholding approach (28). The CCONH experiment and ^1^H-^15^N HSQC spectra were recorded linearly and were processed with NMRpipe (29). All spectra were analyzed with CcpNmr (30).

##### Heteronuclear Single Quantum Coherence Spectroscopy (^1^H-^15^N HSQC) Titration

Titration of 50 μm ^15^N labeled A-Fabp was carried out in NMR titration buffer with increasing concentrations of m^sol^Cgi-58 (0, 10, 25, 50, 100, and 200 μm). ^1^H-^15^N HSQC spectra were recorded after each titration step and compared in CcpNmr (30). Titration of A-Fabp with m^sol^Cgi-58 resulted in the loss of peak intensities and disappearance of peaks at early points of the titration. To analyze the interaction of A-Fabp with Cgi-58, peak intensities in the ^1^H-^15^N HSQC spectrum of A-Fabp recorded in the presence of 100 μm m^sol^Cgi-58 were compared with peak intensities in the ^1^H-^15^N HSQC spectrum of A-Fabp acquired in absence of m^sol^Cgi-58. Those residues, for which the corresponding peak intensities in the ^1^H-^15^N HSQC spectrum experienced a reduction larger than twice the standard deviation compared with the reference spectrum, were mapped on the surface of an A-Fabp crystal structure (Protein Data Bank code 3Q6L).

##### Solid Phase Assay

96-Well polystyrene plates (MaxiSorp, Nalgen Nunc Int., Rochester, NY) were coated overnight with 3 μg of purified Fabp or 1 μg of purified Atgl (as described in Ref. 10) per well. Unspecific binding sites were blocked with 5% (w/v) delipidated BSA (Sigma) dissolved in TBS (50 mm Tris, pH 8.0, 150 mm NaCl) for 2 h. Cell extracts (10–60 μg of total protein) containing equal amounts of His-tagged proteins supplemented with 2% BSA and 0.05% Tween 20 were added and incubated overnight. The plate was rinsed three times with TBS containing 0.05% Tween 20 and incubated with mouse anti-His antibody (GE Healthcare) in the same buffer containing 0.5% delipidated BSA for 1 h. The plate was again washed three times and incubated with the secondary sheep horseradish peroxidase-conjugated (Hrp) anti-mouse antibody (GE Healthcare) in the same buffer as the primary antibody for 1 h. After three final washing steps, antibody binding was visualized with tetramethylbenzidine as chromogenic substrate, and absorbance was measured at 450 nm with 620 nm as reference wavelength. The corresponding Western blots were performed according to standard procedures using mouse anti-His antibody (1:5000, GE Healthcare) and anti-mouse Cy3-conjugated secondary antibody (1:1000, GE Healthcare). Fluorescence was detected in a Typhoon instrument (GE Healthcare), and signal intensities were quantified by densitometry within a linear range.

##### Co-immunoprecipitation

Gonadal WAT from an overnight fasted C57BL/6 mouse was homogenized in IP/lysis buffer (50 mm Tris, pH 7.4, 150 mm NaCl, 1% Nonidet P-40, 1 mm EDTA, 20 μg/ml leupeptin, 2 μg/ml antipain, 1 μg/ml pepstatin, phosphatase inhibitor 2 and 3, Sigma) in a pre-chilled 1-ml Dounce tissue grinder (Wheaton, Millville, NJ). After centrifugation at 20,000 × *g* and 4 °C for 30 min, the infranatant was collected, and an aliquot equaling 700 μg of total protein was incubated with 5 μg of monoclonal antibodies directed against A-Fabp (ab 81605, Abcam, Cambridge, UK), Cgi-58 (H00051099-M01, Abnova, Taipei, Taiwan), or Gfp as negative control (sc-9996, Santa Cruz Biotechnology, Dallas, TX) overnight at 4 °C. Antibody-antigen complexes were precipitated by incubation with 50 μl of equilibrated protein A/G-Sepharose (Thermo Scientific, Waltham) for 2 h and extensively washed with IP/Lysis buffer. Bound proteins were eluted by boiling in SDS sample buffer (50 mm Tris, pH 6.8, 100 mm dithiothreitol, 2% SDS, 10% glycerin, 0.05% bromphenol blue) and analyzed by Western blotting using the aforementioned antibodies.

##### Microscale Thermophoresis

Microscale thermophoresis analyses were carried out with a Monolith NT.115 instrument (Nano Temper, Munich, Germany). Purified Gst-tagged Cgi-58 (10 μm) was covalently linked to the fluorescent label NT-495 by NHS coupling. Increasing concentrations (8 nm to 250 μm) of unlabeled Strep-tagged A-Fabp in 100 mm Tris, pH 8.0, 150 mm NaCl, 1 mm EDTA, 0.05% Tween 20 were titrated against constant amounts of labeled Cgi-58. Standard treated capillaries (K002 Monolith NT.115) were loaded with the samples, and all measurements were performed in duplicate. Data evaluation was performed with the Monolith software (Nano Temper, Munich, Germany).

##### Preparation of Cell and Tissue Extracts

COS-7 cells, transfected or co-transfected with expression vectors encoding Atgl, Cgi-58, and/or A-Fabp (as described above), were disrupted by sonication (Virsonic 475, Virtis, Gardiner, NJ) in solution A. Murine gonadal WAT was homogenized in the same buffer using an Ultra Turrax® homogenizer (Ika GmbH, Staufen, Germany), and the lysates were centrifuged at 20,000 × *g* for 30 min. The infranatants were collected and used for the subsequent assays.

##### Assay for TG Hydrolase Activity

Radiolabeled TG substrate was prepared by emulsifying 1.67 mm triolein (containing 40,000 cpm/nmol [^3^H]triolein as tracer) and 45 μm phosphatidylcholine/phosphatidylinositol (3:1) in 100 mm potassium phosphate buffer, pH 7.0, by sonication (Virsonic 475, Virtis, Gardiner, NJ) as described previously (31). To determine TG hydrolase activity, 10–40 μg of respective cell or tissue extracts in a total volume of 100 μl were either preincubated with 25–100 μm A-Fabp inhibitor BMS309403 (32), 20 μm Atglistatin (33), and/or 10 μm Hsl-inhibitor 76-0079 (NNC 0076-0000-0079, Novo Nordisk, Denmark) or DMSO as control. 100 μl of the TG substrate were added and incubated for 1 h in a 37 °C shaking water bath. Released FAs were extracted with 3.25 ml of methanol/chloroform/heptane 10:9:7 and 1 ml of 0.1 m K_2_CO_3_. After centrifugation at 800 × *g* for 15 min, radioactivity of the aqueous phase was determined by liquid scintillation counting. The inhibitor BMS309403 was synthesized as described in Sulsky *et al.* (34).

##### Firefly Luciferase Gene Transactivation Assay

COS-7 cells were seeded in 24-well plates and co-transfected with expression plasmids using TurboFect (Thermo Scientific). Control cells were co-transfected with pPPRE-Luc, a Ppar-responsive expression vector of *Photinus pyralis* firefly luciferase, and pRLL-Luc encoding *Renilla reniformis* luciferase under the control of a constitutive promoter (both plasmids were kind gifts from B. Steals, University of Lille, France). To determine the impact of Atgl, Cgi-58, and/or Fabps on Ppar activation, cells were additionally transfected with expression vectors encoding these proteins. The total DNA amount was kept constant for all transfections by addition of empty vector-DNA. Luciferase activities were measured after passive cell lysis using the Dual-Luciferase® Reporter assay system (Promega, Madison, WI) in a GloMax-Multi+ Microplate Multimode Reader (Promega). Firefly luminescence units were divided by *Renilla* lumincescence units to normalize to transfection efficiency. Signals of control transfections (cells transfected with pPPRE-Luc and PLL-Luc) were set to 100%, and all other transfections were expressed relative to it.

##### Statistical Analysis

Statistical significance was determined by the Student's unpaired *t* test (two-tailed). Group differences were considered significant for *p* < 0.05 (*), *p* < 0.01 (**), and *p* < 0.001 (***). Data are presented as mean ± S.D.

## Results

### 

#### 

##### A-Fabp and Some Fabp Isoforms Bind to Cgi-58

**FIGURE 1.**
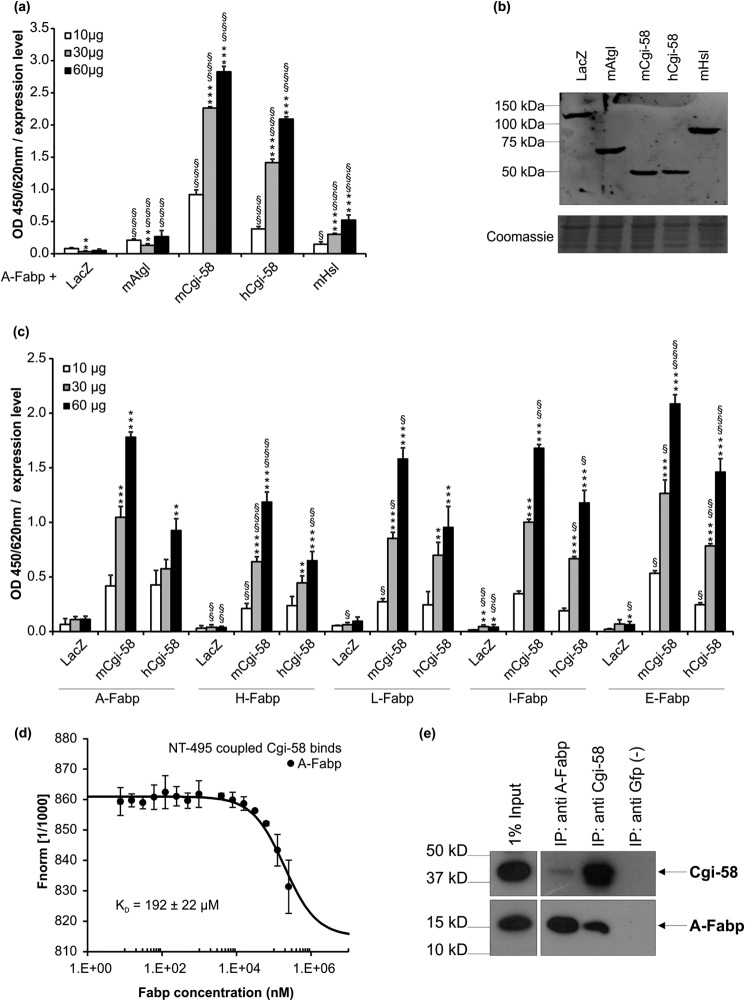
**A-Fabp interacts with Cgi-58.** *a,* in solid phase assays, polystyrene plates were coated with purified A-Fabp and incubated with lysates of COS-7 cells transfected with plasmids encoding His-tagged murine Atgl, murine or human Cgi-58, murine Hsl, and as control β-galactosidase (LacZ). Bound proteins were detected using anti-His primary and Hrp-conjugated secondary antibody. Plates were developed using tetramethylbenzidine as substrate, and the absorbance was measured at 450/620 nm. Absorbance values were normalized to the relative expression levels of prey proteins determined by densitometry. *b,* Western blot analysis of respective cell lysates using anti-His primary and Cy3-conjugated secondary antibody. Fluorescence was quantified in a typhoon instrument. *c,* Cgi-58 interacts with various Fabp isoforms. Polystyrene plates were coated with purified A-, H-, L-, I-, or E-Fabp and incubated with COS-7 cell lysates (10, 30, and 60 μg total protein) containing His-tagged murine Cgi-58, human Cgi-58, and as control LacZ, expressed at comparable levels. Binding of proteins was detected using anti-His primary and Hrp-conjugated secondary antibody. *d,* A-Fabp/Cgi-58 interaction demonstrated by microscale thermophoresis. Ten μmol of purified and fluorescently labeled Cgi-58 was titrated against increasing amounts of unlabeled A-Fabp and fluorescence distribution inside the capillary determined using the Monolith NT.115. *F*_norm_, normalized fluorescence. *e,* co-immunoprecipitation of endogenous A-Fabp and Cgi-58. White adipose tissue from overnight fasted mice was lysed and incubated with antibodies directed against A-Fabp, Cgi-58, or Gfp as negative control. Antibody/antigen complexes were precipitated using protein A/G-Sepharose and analyzed together with the lysate (= input) for the presence of antigens by Western blotting. Data are shown as mean ± S.D. (*n* = 4) and are representative of three independent experiments. Statistical difference was determined as compared with LacZ control (*, *p* < 0.05; **, *p* < 0.01; ***, *p* < 0.001) and of 60 and 30 μg *versus* 10 μg of each lysate (§, *p* < 0.05; §§, *p* < 0.01; §§§, *p* < 0.001).

To identify potential binding partners of Cgi-58, we performed a preliminary label-transfer screen using adipose tissue lysates and label-transfer reagent-modified Cgi-58 as bait. The initial exploratory approach revealed a large number of potential interaction partners summarized in Table 1. The most frequently detected Cgi-58-binding protein in MS analysis was A-Fabp as well as another Fabp family member, namely E-Fabp. To confirm and substantiate this initial observation of an interaction between Cgi-58 and A-Fabp, we applied four independent methods as follows: solid phase assays, microscale thermophoresis, co-immunoprecipitation, and NMR titration. For the solid phase assays, purified A-Fabp was immobilized on microtiter plates and incubated with COS-7 cell lysates containing recombinant murine or human Cgi-58, murine Atgl, Hsl, or LacZ as negative control. Specific binding of His-tagged Cgi-58 to A-Fabp was detected in a sandwich assay using His tag-specific antibodies and Hrp-coupled secondary antibodies against mouse IgG. Both mouse and human Cgi-58 bound to A-Fabp in a dose-dependent manner (Fig. 1*a*). Hsl also dose-dependently bound to A-Fabp confirming previous observations (35–37). Atgl or LacZ protein did not bind to A-Fabp. All solid phase assays contained similar amounts of recombinant protein as estimated by Western blotting signal intensities (Fig. 1*b*). Solid phase assays with other members of the Fabp family revealed specific binding of mouse and human Cgi-58 to H-Fabp, I-Fabp, L-Fabp, and E-Fabp (Fig. 1*c*).

**TABLE 1:** **List of potential Cgi-58 interaction partners identified by label transfer experiments with subsequent nano-LC/MS-MS analysis** Purified Gst-tagged Cgi-58 was labeled with the heterobifunctional cross-linker Sulfo-SBED and incubated with murine WAT lysates to label interacting proteins with a biotin tag. After affinity purification, the interacting proteins were separated by SDS-PAGE, tryptically digested in gel, and analyzed with nano-LC-MS/MS. Proteins found in the respective samples including the NCBI accession numbers, their molecular masses, the number of identified distinct peptides, the Spectrum Mill protein score, and the percentage of amino acid coverage are given in the table. The database used for re-analysis of the raw data was the “mouse” subset of the “SwissProt” database, downloaded on 7.1.2014 from “ncbi.nih.gov” (456,996 total entries). Database searching was performed with the “MS/MS Search” feature of the “Agilent-Spectrum Mill” software (revision 2.7). Acceptance parameter: individual peptide score: min 10; protein score: min 20, and minimum number of distinct peptides: 2. Keratin was excluded from the list.

Spectra	Distinct peptides	Distinct summed MS/MS search score	% amino acid coverage	Protein mass	Species	Database accession no.	Protein name
				*Da*			
**Sample 1**							
33	6	90.5	20	29,366.4	Mouse	P16015.3	Carbonic anhydrase 3
6	5	70.35	22	24,870.8	Mouse	O08709.3	Peroxiredoxin-6
6	4	57.09	14	25,970.2	Mouse	P10649.2	Glutathione *S*-transferase Mu 1
6	4	51.08	5	68,693	Mouse	P07724.3	Serum albumin; Flags, precursor
3	3	46.96	11	27,623.4	Mouse	Q9DCW4.3	Electron transfer flavoprotein subunit β; short = β-ETF
6	3	46.64	10	28,086.6	Mouse	Q9CQV8.3	14-3-3 protein β/α
3	3	45.14	12	23,013.9	Mouse	P14602.3	Heat shock protein β-1; short = HspB1
4	3	43.9	12	28,832.1	Mouse	Q9DBJ1.3	Phosphoglycerate mutase 1
3	2	29.75	6	32,191.9	Mouse	P17751.4	Triosephosphate isomerase; short = TIM
**3**	**2**	**29.15**	**18**	**14,650**	**Mouse**	**P04117.3**	**Fatty acid-binding protein, adipocyte**
4	2	29.09	7	30,615.7	Mouse	Q00623.2	Apolipoprotein A-I; short = Apo-AI; short = ApoA-I
3	2	26.78	7	26,468.9	Mouse	Q9WTP6.5	Adenylate kinase 2, mitochondrial; short = AK 2
4	2	26.28	7	27,679.8	Mouse	Q03401.1	Cysteine-rich secretory protein 1; short = CRISP-1
4	2	24.81	4	53,951.8	Mouse	Q2KHK6.2	Gasdermin-C2
3	2	23.57	1	217,752.2	Mouse	Q6PDQ2.1	Chromodomain-helicase-DNA-binding protein 4; short = CHD-4
3	2	23.4	3	33,267.1	Mouse	Q8R2Z0.2	Protein FAM132A; Flags: precursor
4	2	22.66	2	117,772.7	Mouse	Q9JHR7.1	Insulin-degrading enzyme
3	2	20.89	0	833,642.4	Mouse	Q91ZU6.1	Dystonin
3	2	20.85	8	37,549	Mouse	O35864.3	COP9 signalosome complex subunit 5; short = SGN5; short = signalosome subunit 5
3	2	20.64	0	292,121	Mouse	Q8C170.2	Unconventional myosin-IXa
2	2	20.22	0	390,6506.8	Mouse	A2ASS6.1	Titin
2	2	20.09	0	282,962.5	Mouse	Q5DU37.2	Zinc finger FYVE domain-containing protein 26

**Sample 2**							
16	8	117.71	51	15,878.3	Mouse	P02089.2	Hemoglobin subunit β-2
**20**	**5**	**87.34**	**34**	**14,650**	**Mouse**	**P04117.3**	**Fatty acid-binding protein, adipocyte**
26	5	78.53	31	15,085.2	Mouse	P01942.2	Hemoglobin subunit α
8	3	53.7	15	22,176.6	Mouse	P35700.1	Peroxiredoxin-1
4	3	50.67	11	29,366.4	Mouse	P16015.3	Carbonic anhydrase 3
4	3	47.96	24	13,992.3	Mouse	Q8CGP2.3	Histone H2B type 1-P
4	4	46.9	0	3,906,506.8	Mouse	A2ASS6.1	Titin
3	3	46.02	18	20,830.5	Mouse	P70296.3	Phosphatidylethanolamine-binding protein 1; short = PEBP-1
9	3	45.27	26	14,161.6	Mouse	Q8CGP5.3	Histone H2A type 1-F
5	3	44.97	17	17,971.4	Mouse	P17742.2	Peptidyl-prolyl cis-trans isomerase A; short = PPIase A
3	2	37.14	13	21,897.6	Mouse	P99029.2	Peroxiredoxin-5, mitochondrial
**4**	**2**	**29.5**	**13**	**15,137.5**	**Mouse**	**Q05816.3**	**Fatty acid-binding protein, epidermal**
3	3	28.28	22	36,347.9	Mouse	Q8C0D0.1	Probable tRNA pseudouridine synthase 1
9	2	27.38	3	79,574.8	Mouse	Q8R322.2	Nucleoporin GLE1
3	3	24.98	0	55,0837	Mouse	Q9QYX7.4	Protein piccolo
3	2	24.85	14	22,395.6	Mouse	Q9WVA4.4	Transgelin-2
3	2	23.5	1	245,759.3	Mouse	Q8BZN6.3	Dedicator of cytokinesis protein 10
3	2	22.18	1	281,223	Mouse	Q8BTM8.5	Filamin-A; short = FLN-A
2	2	22.02	0	519,211.7	Mouse	A2ARV4.1	Low-density lipoprotein receptor-related protein 2; short = LRP-2
3	2	21.65	6	40,489.2	Mouse	P08752.5	Guanine nucleotide-binding protein G_i_ subunit α-2
4	2	21.08	1	157,204.6	Mouse	O09053.3	Werner syndrome ATP-dependent helicase homolog
2	2	20.17	2	103,938	Mouse	Q6P9P8.1	DENN domain-containing protein 2C

**Sample 3**							
10	6	101.96	55	15,840.3	Mouse	P02088.2	Hemoglobin subunit β-1
11	5	74.76	31	15,085.2	Mouse	P01942.2	Hemoglobin subunit α
6	5	57.86	28	17,971.4	Mouse	P17742.2	Peptidyl-prolyl cis-trans isomerase A; short = PPIase A
**3**	**2**	**35.84**	**16**	**14,650**	**Mouse**	**P04117.3**	**Fatty acid-binding protein, adipocyte**
3	2	35.5	14	22,395.6	Mouse	Q9WVA4.4	Transgelin-2
4	2	33.14	19	13,994.3	Mouse	Q9D2U9.3	Histone H2B type 3-A
4	2	32.25	21	14,161.6	Mouse	Q8CGP5.3	Histone H2A type 1-F
2	2	31.95	15	18,559.7	Mouse	P18760.3	Cofilin-1
2	2	31.74	11	20,830.5	Mouse	P70296.3	Phosphatidylethanolamine-binding protein 1; short = PEBP-1
**3**	**2**	**31.41**	**13**	**15,137.5**	**Mouse**	**Q05816.3**	**Fatty acid-binding protein, epidermal**
3	3	30.13	16	61,422.6	Mouse	Q61207.2	Sulfated glycoprotein 1; short = SGP-1
5	2	29.13	11	14,865.9	Mouse	P16045.3	Galectin-1; short = Gal-1
3	3	28.14	0	3,906,506.8	Mouse	A2ASS6.1	Titin
4	2	25.17	13	15,942.8	Mouse	P08228.2	Superoxide dismutase (CuZn)
4	2	24.57	17	10,962.8	Mouse	Q64433.2	10 kDa heat shock protein, mitochondrial; short = Hsp10
7	2	23.08	3	167,141.5	Mouse	Q925J9.2	Mediator of RNA polymerase II transcription subunit 1
5	2	23	3	79,574.8	Mouse	Q8R322.2	Nucleoporin GLE1
2	2	22.91	11	32,970.4	Mouse	O35075.1	Down syndrome critical region protein 3 homolog
2	2	22.74	4	62,700.3	Mouse	Q9JHU2.1	Palmdelphin
2	2	22.06	6	40,489.2	Mouse	P08752.5	Guanine nucleotide-binding protein G_i_ subunit α-2
3	2	21.9	1	244,562.5	Mouse	A2AGH6.1	Mediator of RNA polymerase II transcription subunit 12
3	2	21.29	2	124,935	Mouse	Q6A025.2	Protein phosphatase 1 regulatory subunit 26
10	2	20.4	1	157,204.6	Mouse	O09053.3	Werner syndrome ATP-dependent helicase homolog
2	2	20.07	3	96294.8	Mouse	Q8C9H6.1	Striatin-interacting proteins 2
2	2	20	0	392,324.8	Mouse	Q9JJN2.1	Zinc finger homeobox protein 4

**Sample 4**							
61	11	205.71	88	15,840.3	Mouse	P02088.2	Hemoglobin subunit β-1
**32**	**9**	**170.54**	**60**	**14,650**	**Mouse**	**P04117.3**	**Fatty acid-binding protein, adipocyte**
14	7	117.46	30	17,971.4	Mouse	P17742.2	Peptidyl-prolyl cis-trans isomerase A; short = PPIase A
8	6	114.55	29	29,366.4	Mouse	P16015.3	Carbonic anhydrase 3
44	6	103.08	42	15,085.2	Mouse	P01942.2	Hemoglobin subunit α
8	4	73.3	28	21,897.6	Mouse	P99029.2	Peroxiredoxin-5, mitochondrial
4	4	61.97	47	13,159.6	Mouse	Q9Z0F7.1	γ-Synuclein
6	3	60	25	15,942.8	Mouse	P08228.2	Superoxide dismutase (CuZn)
7	4	59.11	30	14,865.9	Mouse	P16045.3	Galectin-1; short = Gal-1
8	3	56.68	28	20,830.5	Mouse	P70296.3	Phosphatidylethanolamine-binding protein 1; short = PEBP-1
4	3	50.09	25	15,776	Mouse	P07309.1	Transthyretin
5	3	50	4	68,693	Mouse	P07724.3	Serum albumin; Flags: precursor
**6**	**3**	**48.45**	**14**	**15,137.5**	**Mouse**	**Q05816.3**	**Fatty acid-binding protein, epidermal**
4	4	39.37	0	3,906,506.8	Mouse	A2ASS6.1	Titin
3	2	36.31	29	11,082.8	Mouse	P50543.1	Protein S100-A11
3	2	36.14	3	82,550.5	Mouse	P0CG50.2	Polyubiquitin-C; contains: ubiquitin; contains: ubiquitin-related 1; contains: ubiquitin-related 2; Flags: precursor
3	2	33.74	21	18,559.7	Mouse	P18760.3	Cofilin-1
3	2	31.27	19	14,255.5	Mouse	P52760.3	Ribonuclease UK114
4	3	26.9	6	113,100.4	Mouse	P11103.3	Poly [ADP-ribose] polymerase 1; short = PARP-1
2	2	25.62	23	10,962.8	Mouse	Q64433.2	10 kDa heat shock protein, mitochondrial; short = Hsp10

Next, microscale thermophoresis, a novel method for immobilization-free analysis of protein/protein interactions, was used to assess the Cgi-58/A-Fabp interaction *in vitro*. Thermophoresis is the directed movement of proteins in response to a temperature gradient and depends on particle charge, size, conformation, and hydration state. Thus, under constant buffer conditions thermophoresis of unbound proteins typically differs from the thermophoresis of proteins bound to interaction partners. Microscopic temperature gradients are generated by an infrared laser focused on a capillary. Thermophoretic movement of a fluorescently labeled protein is then measured by monitoring the fluorescence distribution. When fluorescently labeled Cgi-58 was titrated against increasing amounts of purified A-Fabp, its thermophoretic movement markedly changed indicating protein/protein interaction (Fig. 1*d*). Calculation of an equilibrium dissociation constant revealed a *K_d_* of 192 ± 22 μm. According to Ozbabacan *et al.*, (7) a *K_d_* value in this micromolar range is commonly found for weak and transient interactions.

To further corroborate the Cgi-58/A-Fabp interaction, we performed co-immunoprecipitation experiments with murine WAT lysates. A monoclonal A-Fabp antibody bound to protein A/G-Sepharose co-precipitated Cgi-58. Conversely, a protein A/G-Sepharose-bound Cgi-58 antibody co-precipitated A-Fabp (Fig. 1*e*). Neither A-Fabp nor Cgi-58 were detected when the lysates were incubated with a protein A/G-Sepharose-bound Gfp antibody as negative control. These data show that the interaction between Cgi-58 and A-Fabp can be detected with endogenous proteins under close-to-physiological conditions.

**FIGURE 2.**
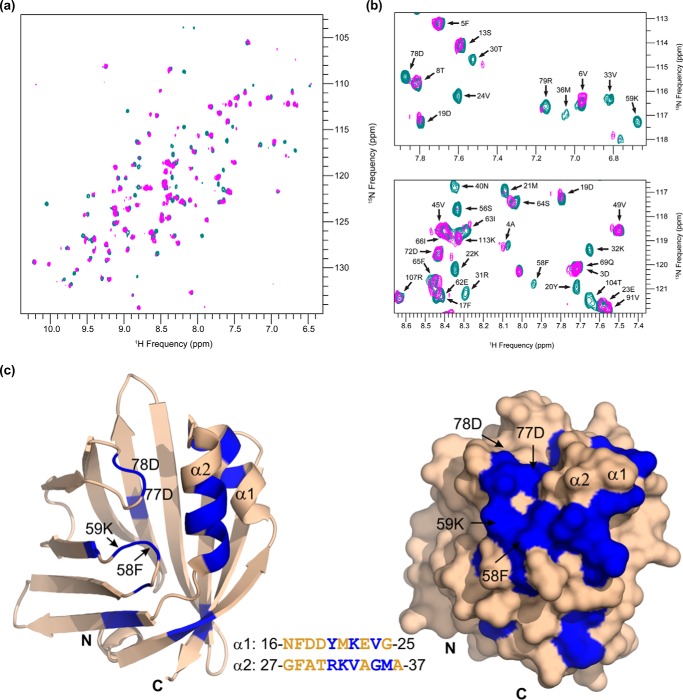
**Mapping of the A-Fabp/mCgi-58 binding interface by NMR titrations.** Two-dimensional ^1^H-^15^N HSQC experiments result in a spectrum showing peaks (with their intensity values depicted in contour plots) for each N-H pair. Each peak represents a ^1^H-^15^N pair, with the respective ^1^H and ^15^N resonance frequencies. Consequently, the resulting peaks correspond mostly to backbone amide groups of A-Fabp. Assignment of the peaks to individual backbone residues was carried out upon analysis of triple resonance experiments. *a,* ^1^H-^15^N HSQC spectra were recorded for 50 μm ^15^N-labeled A-Fabp in free form (*cyan*) and in complex with 100 μm m^sol^Cgi-58 (*magenta*). Protein/protein interaction induces changes in the chemical environment of ^1^H-^15^N pairs in the binding interface and results in intensity perturbations of the peaks corresponding to the involved residues. *b,* selected regions of the spectrum show a subset of peaks representing free A-Fabp in *cyan* and A-Fabp complexed with mCgi-58 (*magenta*). Intensity changes in peaks corresponding to residues involved in the A-Fabp/mCgi-58 interface ranged from a significant decline of intensity to full disappearance of the *magenta* peaks. *c,* ribbon and surface representations of the three-dimensional structure of A-Fabp (Protein Data Bank code 3Q6L). Residues in *blue* correspond to residues experiencing a significant decrease in peak intensity upon addition of m^sol^Cgi-58. The *inset* shows the primary sequence of helix α1 and helix α2 with the affected residues highlighted in *blue*. Structure representations were prepared with PyMOL (PyMOL Molecular Graphics System, Version 1.5.0.4 Schrödinger, LLC.).

Finally, we confirmed the Cgi-58/A-Fabp interaction by NMR titration experiments using a more soluble but functional variant of Cgi-58 termed m^sol^Cgi-58 (38). A two-dimensional heteronuclear single quantum coherence spectrum (^1^H-^15^N HSQC) was recorded with a sample of ^15^N-labeled A-Fabp. The observed peaks (depicted as contour plots) in the spectrum correspond to bonded ^15^NH pairs, mainly present in the protein backbone. The peak dispersion of the ^1^H-^15^N HSQC spectrum of ^15^N-labeled A-Fabp indicated a folded protein with a high amount of β-sheet content as expected from the three-dimensional structure. The chemical shifts are very sensitive to the chemical environment of each ^15^NH pair. Therefore, addition of an interacting protein leads to marked changes of the chemical shift and/or the intensities of the respective peaks. As seen in Fig. 2*a*, addition of unlabeled m^sol^Cgi-58 to ^15^N-labeled A-Fabp led to significant changes in the two-dimensional spectrum indicating interaction of these two proteins (Fig. 2*a*).

##### A-Fabp Binding to Cgi-58 Involves Amino Acid Residues of the Helix-Loop-Helix Cap Region

Next, triple resonance experiments were recorded and analyzed to assign the observed NMR resonances to individual amino acids. Residues most affected upon titration with m^sol^Cgi-58 included Lys-10, Leu-11, Tyr-20, Lys-22, Val-24, Arg-31, Lys-32, Val-33, Gly-35, Met-36, Met-41, Ile-42, Ser-56, Phe-58, Lys-59, Thr-61, Asp-77, Asp-78, His-94, Leu-114, Val-119, and Tyr-129 (Fig. 2*b*). Mapping these residues onto the known three-dimensional structure of A-Fabp (Protein Data Bank code 3Q6L) revealed that a region encompassing helix α1 and α2 and two spatially adjacent loops (including Phe-58, Lys-59, Asp-77, and Asp-78) very likely represent the binding interface between Cgi-58 and A-Fabp (Fig. 2*c*).

**FIGURE 3.**
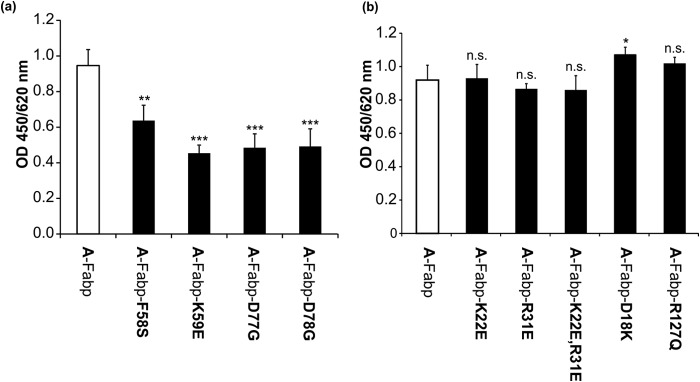
**Binding of Cgi-58 to various mutant A-Fabp variants.** *a,* in solid phase assays, polystyrene plates were coated with 3 μg of purified wild type A-Fabp or the mutant variants A-Fabp^F58S^, A-Fabp^K59E^, A-Fabp^D77G^, and A-Fabp^D78G^ and incubated with COS-7 lysates (60 μg of total protein) containing murine His-Cgi-58. Bound proteins were detected using anti-His primary and Hrp-conjugated secondary antibody. Plates were developed using tetramethylbenzidine as substrate, and the absorbance was measured at 450/620 nm. *b,* solid phase assays detecting the binding of wild type A-Fabp or the mutant variants A-Fabp^D18K^, A-Fabp^K22E^, and A-Fabp^R31E^ as well as the double mutant A-Fabp^K22E,R31E^ to murine His-Cgi-58 expressed in COS-7 cells. Each mutant variant features an exchange in one or two amino acids involved in binding of Hsl to A-Fabp. The mutant variant A-Fabp^R127Q^, which is not capable of binding FAs, was also tested for binding to Cgi-58. Data are shown as mean ± S.D. (*n* = 4) and are representative of three independent experiments. Statistical difference was determined as compared with A-Fabp control (*n.s.*, nonsignificant; *, *p* < 0.05; **, *p* < 0.01; ***, *p* < 0.001).

To investigate whether these amino acids are crucial for A-Fabp interaction with Cgi-58, we generated mutant A-Fabp variants and tested their binding to Cgi-58 in solid phase assays. A-Fabp^F58S^, A-Fabp^K59E^, A-Fabp^D77G^, and A-Fabp^D78G^ showed 20–50% decreased binding to Cgi-58 as compared with wild type A-Fabp (Fig. 3*a*), indicating that residues Phe-58, Lys-59, Asp-77, and Asp-78 are involved in the binding of A-Fabp to Cgi-58. Decreased but not abolished binding suggests that none of the residues are indispensable *per se*. We also tested A-Fabp mutant variants with amino acid exchanges at Asp-18, Lys-22, and Arg-31. These residues, together with Asp-19, constitute a charged amino acid quartet that has been shown to be essential for the binding of A-Fabp to Hsl (37). However, the mutant variants A-Fabp^D18K^, A-Fabp^K22E^, A-Fabp^R31E^, and A-Fabp^K22E,R31E^ exhibited similar binding to Cgi-58 as WT A-Fabp (Fig. 3*b*) indicating that these amino acid residues may not be required for the A-Fabp/Cgi-58 interaction.

To test whether A-Fabp binding to Cgi-58 depends on its loading status with FAs, we generated the mutant variant A-Fabp^R127Q^, which is unable to bind FAs (24). Solid phase assays revealed similar binding efficiencies of the mutant and WT A-Fabp to murine Cgi-58 indicating that the interaction occurs independently of the FA loading status of A-Fabp (Fig. 3*b*). In contrast, the interaction of A-Fabp with Hsl occurs only when A-Fabp is loaded with a FA (36).

##### A-Fabp Binds to the C-terminal Region of Cgi-58 and Does Not Bind to Atgl

**FIGURE 4.**
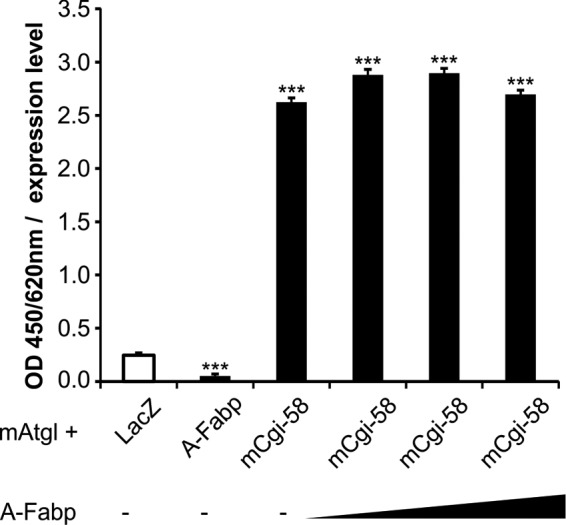
**A-Fabp does not compete with Atgl for Cgi-58 binding.** In solid phase assays, polystyrene plates were coated with 1 μg of purified Atgl and incubated with bacterial lysates (60 μg of total protein) containing LacZ, A-Fabp, or mCgi-58 together with increasing amounts of purified Strep-A-Fabp (0.5-, 1-, or 5-fold molar excess compared with Atgl). Bound proteins were visualized by anti-Strep primary and Hrp-conjugated secondary antibody. Plates were developed using tetramethylbenzidine as substrate, and the absorbance was measured at 450/620 nm. Data are shown as mean ± S.D. (*n* = 4) and are representative of three independent experiments. Statistical difference was determined as compared with LacZ control (***, *p* < 0.001).

Next, we asked whether binding of A-Fabp to Cgi-58 affects the interaction of Cgi-58 with Atgl. In competitive solid phase assays, partially purified Atgl was adsorbed to polystyrene plates and incubated with bacterial lysates containing Strep-tagged Cgi-58 together with increasing amounts of purified A-Fabp. After excessive washing, the amount of retained Cgi-58 was determined using a Strep-tag-specific antibody and an Hrp-coupled secondary antibody against mouse IgG. As shown in Fig. 4, the addition of A-Fabp did not interfere with the binding of Cgi-58 to Atgl at any concentration tested. As assumed, LacZ as a negative control failed to bind to Atgl. This result implicates that Atgl and A-Fabp binding to Cgi-58 occurs at different noncompetitive sites. Consistent with our data above (Fig. 1*a*), we could not detect a direct interaction between Atgl and A-Fabp.

**FIGURE 5.**
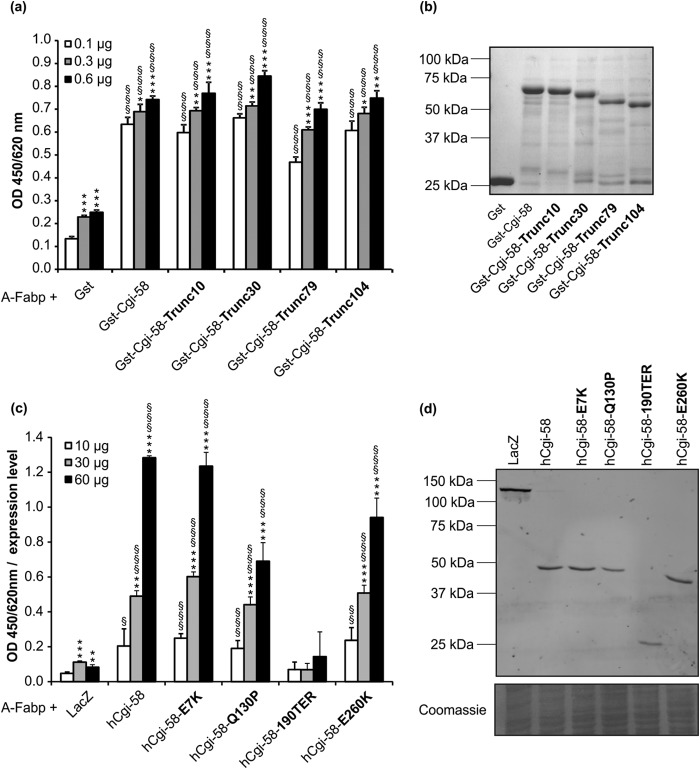
**Identification of Cgi-58 regions involved in binding to A-Fabp.** *a,* in solid phase assays, polystyrene plates were coated with purified A-Fabp and increasing amounts of purified Gst, Gst-Cgi-58, or N-terminally truncated Cgi-58 variants. Binding of proteins was detected using anti-Gst primary and Hrp-conjugated secondary antibody. Plates were developed using tetramethylbenzidine as substrate, and the absorbance was measured at 450/620 nm. *b,* SDS-PAGE of purified Gst, Gst-Cgi-58, and N-terminally truncated Cgi-58 variants after Coomassie Brilliant Blue staining. *c,* solid phase assay detecting the interaction between purified A-Fabp and His-tagged human Cgi-58, the naturally occurring mutant variants of human Cgi-58, and LacZ (control) contained in lysates of transfected COS-7 cells. *d,* Western blot analysis of respective cell lysates using anti-His primary and Cy3-conjugated secondary antibody. Statistical difference was determined as compared with Gst/LacZ control (*, *p* < 0.05; **, *p* < 0.01; ***, *p* < 0.001) and of 0.6/60 and 0.3/30 μg *versus* 0.1/10 μg of each lysate (§, *p* < 0.05; §§, *p* < 0.01; §§§, *p* < 0.001).

To identify potential binding regions in Cgi-58 that interact with A-Fabp, we tested various structural variants of Cgi-58 in solid phase assays. These binding studies revealed that full-length Cgi-58 and N-terminally truncated Cgi-58 variants lacking amino acids between 1 and 10 to 104 of the N terminus (Cgi-58-N-trunc10, Cgi-58-N-trunc30, Cgi-58-N-trunc79, and Cgi-58-N-trunc104) bound equally well to immobilized A-Fabp (Fig. 5, *a* and *b,* shows a control SDS-PAGE of purified Cgi-58 and the truncated variants), indicating that the first 104 amino acids of Cgi-58 are not involved in A-Fabp binding. Apparently, the mutant Cgi-58 variants are able to bind to A-Fabp despite the fact that they at least partially lose their abilities to stimulate Atgl and to localize to LDs (13).

We also investigated the A-Fabp interaction of Cgi-58 with mutations causative for neutral lipid storage disease also named Chanarin-Dorfman syndrome (10, 39). Consistent with the results of the deletion mutants analyzed above, a C-terminally truncated variant of Cgi-58 carrying a premature stop codon at residue 190 (Cgi-58^190TER^) expressed in COS-7 cells almost entirely lost its ability to bind to immobilized A-Fabp. Cgi-58^Q130P^ and Cgi-58^E260K^, two variants with single amino acid exchanges, showed a 46 and 27% decrease in binding capability to A-Fabp, respectively. The amino acid exchange E7K (Cgi-58^E7K^) did not affect A-Fabp binding (Fig. 5, *c* and *d,* shows a control Western blot of the respective COS-7 cell lysates). Overall, these data demonstrate that Cgi-58 binds to A-Fabp via residues in the C-terminal half of the protein and that binding of Cgi-58 occurs independently of its ability to activate Atgl.

##### Binding of A-Fabp to Cgi-58 Stimulates Atgl-mediated Lipolysis

**FIGURE 6.**
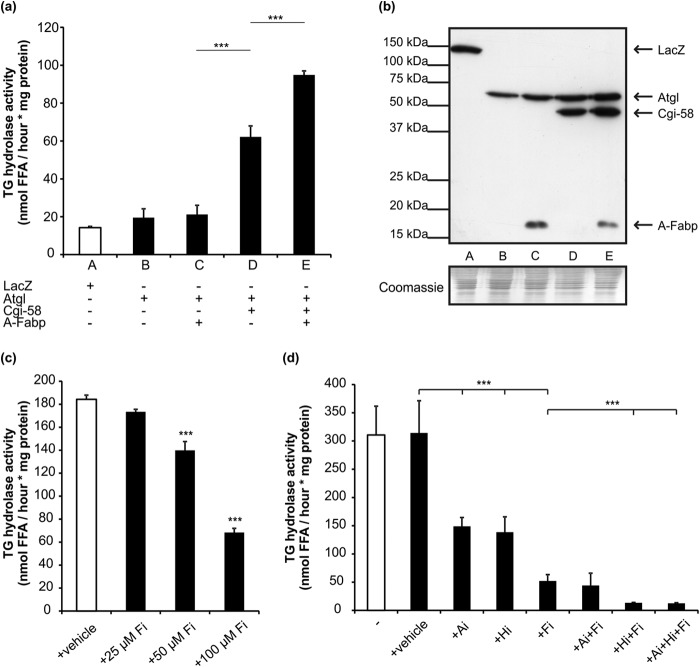
**A-Fabp promotes lipolysis solely in the presence of Cgi-58.** *a,* TG hydrolase activities of cell lysates derived from COS-7 cells expressing His-tagged LacZ (control), Atgl, Cgi-58, and/or A-Fabp were determined by incubating lysates with phospholipid-emulsified triolein substrate containing radiolabeled triolein as tracer. After extraction of free FAs, radioactivity was determined by liquid scintillation counting. *b,* Western blot analysis of respective COS-7 lysates using anti-His primary and Hrp-conjugated secondary antibody. *c,* TG hydrolase activities of lysates prepared from differentiated 3T3-L1 adipocytes. Lysates were incubated with artificial substrate as in *a* in presence of increasing concentrations of the A-Fabp-inhibitor BMS309403 (Fi). *d,* TG hydrolase activities of lysates prepared from murine gonadal WAT. Lysates were incubated with artificial substrate as in *a* in presence of BMS309403 (Fi; 100 μm), the Atgl-inhibitor Atglistatin (Ai; 20 μm), and/or the Hsl inhibitor 76-0079 (Hi; 10 μm). Data are shown as mean ± S.D. (*n* = 3) and are representative of three independent experiments. Statistical difference was determined as compared with control (***, *p* < 0.001).

The impact of A-Fabp on the enzymatic function of Atgl was determined by analyzing neutral lipid hydrolase activities of COS-7 cell lysates overexpressing Atgl, Cgi-58, and/or A-Fabp. Overexpression of Atgl alone led to a 38% increase in TG hydrolase activity compared with samples overexpressing LacZ (negative control). The additional presence of Cgi-58 increased the enzyme activity 3.2-fold, although co-expression of A-Fabp did not increase the TG hydrolase activity. Co-expression of all three proteins, A-Fabp, Atgl, and Cgi-58, led to highest TG hydrolase activities demonstrating that A-Fabp stimulates Atgl-mediated TG hydrolysis in a Cgi-58-dependent manner (Fig. 6, *a* and *b,* shows a control Western blot analysis of the respective lysates).

To examine whether A-Fabp inhibition affects TG hydrolase activities of lysates prepared from differentiated 3T3-L1 adipocytes or murine WAT, we used the A-Fabp inhibitor BMS309403 (Fi) in TG hydrolase activity assays. Fi is known to specifically inhibit FA binding of A-Fabp (32). Addition of Fi to 3T3-L1 cell lysates decreased TG hydrolase activity in a dose-dependent manner. At an inhibitor concentration of 100 μm, TG hydrolase activity was inhibited by 65% (Fig. 6*c*). We also performed similar experiments using murine adipose tissue lysates and the A-Fabp inhibitor (Fi), the Atgl inhibitor Atglistatin (Ai) (33), and/or the Hsl inhibitor 76-0079 (Hi) (10). In lysates of murine adipose tissue, the presence of Fi decreased total TG hydrolase activity by 85% (Fig. 6*d*) suggesting that functional A-Fabp is required for lipase function in adipocytes. The addition of Hi decreased TG hydrolase activity in mouse adipose tissue lysates by 56%. This decrease was further potentiated by the presence of Fi suggesting that Fi inhibits lipolysis independently of Hsl. In contrast, Ai did not diminish the remnant hydrolase activity observed in lysates treated with Fi. Thus, Atgl-mediated lipolysis is not functional when A-Fabp is inhibited.

##### Fabps Promote Ppar Signaling

Haemmerle *et al.* (40) demonstrated that Atgl-mediated lipolysis is required for Pparα function and the expression of Pparα target genes in the heart. A conceivable mechanism linking lipolysis to Ppar activity involves the Fabp-mediated transport of FA from the site of production at LDs to the nucleus, where they act as potential ligands for Ppars (41). To elucidate whether the interaction of Fabps with Cgi-58 affects Ppar activation, we performed luciferase gene transactivation assays in transfected COS-7 cells. The assay was based on a reporter plasmid expressing firefly luciferase under transcriptional control of multiple Ppar-responsive elements (PPRE), which results in the production of luminescence directly proportional to Ppar activity.

**FIGURE 7.**
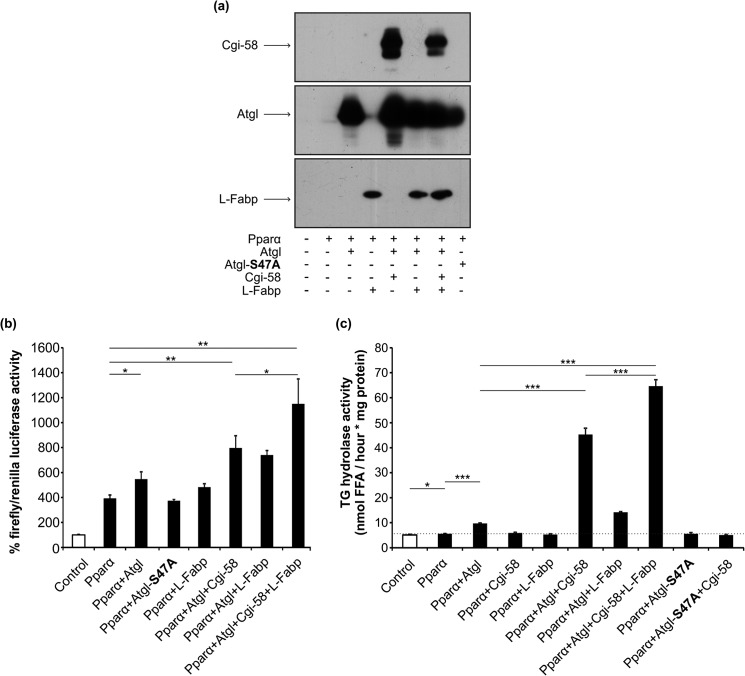
**L-Fabp promotes Pparα signaling.** *a,* Western blot analysis of COS-7 cells that were transfected with a reporter plasmid coding for firefly luciferase under transcriptional control of multiple Ppar-responsive elements (*PPRE*), a plasmid coding for *Renilla* luciferase (transfection control), and a single plasmid or a mixture of plasmids coding for Pparα, Atgl, L-Fabp, Cgi-58, or the catalytically inactive Atgl^S47A^ (as indicated). *b,* for firefly luciferase gene transactivation assays, relative luminescence units of firefly and *Renilla* luciferase were determined in a 24-well plate luminometer and calculated relative to *Renilla* luciferase activities (control values were set to 100%). *c,* TG hydrolase activity (as in Fig. 6) assay and data are shown as mean ± S.D. (*n* = 4) and are representative of three independent experiments. Statistical difference was determined as compared with control (*, *p* < 0.05; **, *p* < 0.01; ***, *p* < 0.001).

Because L-Fabp has been shown to bind to Pparα (42–44), we co-transfected COS-7 cells with the reporter plasmid and various combinations of expression plasmids for Pparα and L-Fabp, Atgl, the enzymatically inactive mutant variant Atgl^S47A^, and Cgi-58. Western blotting analysis revealed similar expression efficiency for the same constructs (Fig. 7*a*). We found that the expression of L-Fabp alone increased Pparα-driven luciferase production by 23%. The additional expression of Atgl and Atgl/Cgi-58 further increased luciferase activity by 54 and 140%, respectively, although the inactive variant of Atgl^S47A^ failed to further induce luciferase activity (Fig. 7*b*). Additionally, we measured TG hydrolase activity and observed a strong correlation between luciferase activities with TG hydrolase activities (Fig. 7*c*).

**FIGURE 8.**
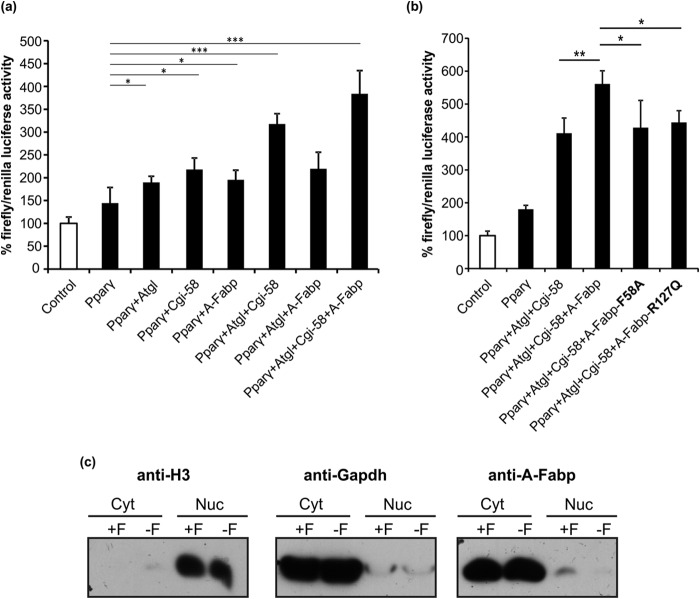
**A-Fabp promotes Pparγ signaling.** *a* and *b,* for firefly luciferase gene transactivation assays, COS-7 cells were transfected with the reporter plasmid coding for firefly luciferase under transcriptional control of multiple PPRE, a plasmid coding for *Renilla* luciferase (transfection control), and a plasmid or a mixture of plasmids coding for Pparγ, Atgl, A-Fabp, Cgi-58, the non-FA binding mutant variant A-Fabp^R127Q^, or the mutant variant A-Fabp^F58A^ incapable of ligand-induced nuclear translocation (as indicated). Relative luminescence units of firefly and *Renilla* luciferase were determined in a 24-well plate luminometer and calculated relative to *Renilla* luciferase activities (control values were set to 100%). *c,* Western blot analysis of cytoplasmic and nuclear fractions obtained from differentiated 3T3-L1 adipocytes under nonstimulated (= basal, −*F*) and forskolin-stimulated (+*F*) conditions. A-Fabp was detected using an antibody specific for A-Fabp and Hrp-conjugated secondary antibody. Purity of the fractions was assessed by Western blotting using anti-histone H3 and anti-Gapdh antibodies, respectively. Data are shown as mean ± S.D. (*n* = 4) and are representative of three independent experiments. Statistical difference was determined as compared with control (*, *p* < 0.05; **, *p* < 0.01; ***, *p* < 0.001).

Similar results were obtained when Pparγ was co-expressed with Atgl, Cgi-58, and A-Fabp in COS-7 cells (Fig. 8*a*). The expression of A-Fabp alone increased Pparγ-driven luciferase production by 36%. The expression of Atgl and Atgl/Cgi-58 increased luciferase activity by 32 and 121%, respectively. Highest firefly luciferase activities were achieved by co-transfection of Pparγ, Atgl, Cgi-58, and A-Fabp, indicating that the presence of A-Fabp further promotes Pparγ activation.

To test whether functional FA binding to A-Fabp was required for the activation of Pparγ, we performed luciferase gene transactivation assays with A-Fabp^R127Q^, a variant unable to bind FAs. This mutant Fabp variant did not further stimulate firefly luciferase activities when co-expressed with Pparγ, Atgl, and Cgi-58 (Fig. 8*b*), indicating that FA binding is a prerequisite for Pparγ-driven reporter gene activation. Similar effects were observed when applying the mutant variant A-Fabp^F58A^ with a defect in ligand-induced nuclear translocation (47). Also, A-Fabp^F58A^ failed to increase firefly luciferase activity upon co-expression with Atgl and Cgi-58, implying that functional nuclear import of ligand-bound A-Fabp is essential for A-Fabp-mediated Pparγ activation (Fig. 8*b*). To further corroborate these findings, we investigated the subcellular distribution of endogenous A-Fabp in response to forskolin-stimulated lipolysis. Therefore, we analyzed localization of A-Fabp in differentiated, nonstimulated (basal), or forskolin-stimulated 3T3-L1 cells by Western blotting (Fig. 8*c*). A-Fabp was predominantly detected in the cytosol under both conditions. Notably, however, the amount of A-Fabp in the nuclear fraction markedly increased upon forskolin stimulation arguing for an increased A-Fabp translocation into the nucleus under lipolytic conditions. Thus, both FA binding and nuclear translocation of A-Fabp are required for Atgl/Cgi-58-mediated Pparγ stimulation.

## Discussion

The identification and characterization of components that constitute a functional lipolysome are essential for a detailed understanding of fat catabolism and energy homeostasis. Several of the recently discovered enzymes and regulatory proteins not only affect lipolysis *per se* but are also linked to other cellular processes such as inflammation, cancer, apoptosis, or cell signaling. For example, Pedf stimulates lipolysis via Atgl (5) but also has anti-angiogenic, neuroprotective, and anti-tumor activities (45). Other examples include the noncompetitive Atgl inhibitor G0s2 (20) that also interacts with OXPHOS complex V leading to increased cellular ATP synthesis (46) or the Atgl-binding partner Gbf-1, which is part of the Arf1/CopI machinery, involved in membrane trafficking pathways (23). Even the indispensable Atgl co-activator Cgi-58 has partially undefined non-Atgl-related functions in liver, skin, and macrophages (47–49). This plurality of factors regulating TG hydrolysis and their additional biochemical functions beyond lipid degradation highlights the close link of lipolysis with other cellular pathways.

In this study, we show that A-Fabp forms a physical complex with the Atgl co-activator Cgi-58, both *in vitro* and in living cells. A-Fabp belongs to a conserved nine-member multigene family of intracellular lipid-binding proteins with ubiquitous tissue distribution. Even though the individual Fabp isoforms exhibit marked differences in ligand affinity, selectivity, and binding mechanisms, all Fabps bind long-chain FAs with high affinity (50, 51). Because this process is essential to prevent (lipo)toxic effects via FA membrane interaction or micelle formation, we propose that A-Fabp/Cgi-58 binding is required to absorb lipolysis-derived FAs. Interestingly, the plant ortholog of Cgi-58 interacts with the ABC transporter 1 (Pxa1), a protein that transports FAs or lipophilic hormone precursors into peroxisomes for metabolic processing (52). We also show that Cgi-58 interacts with five of nine members of the Fabp family suggesting that the Cgi-58/A-Fabp interaction may be relevant in multiple cell types and tissues, a conclusion that is also consistent with the broad tissue expression pattern of both proteins.

The interface responsible for the protein/protein interaction involves the helix α1-turn-helix α2 region of A-Fabp and two adjacent loops. Interestingly, single amino acid exchanges within the two adjacent loops led to significantly decreased binding of A-Fabp to Cgi-58. Because members of the Fabp family are highly related and share similar tertiary structures (53), we assume that the Cgi-58 binding interface is analogous in all Fabp isoforms. Furthermore, the mutant variant of A-Fabp that lacks more than 99% of the FA binding capacity of the wild type protein was still able to bind to Cgi-58 suggesting that FA loading of A-Fabp is not required for the interaction between the two proteins.

Notably, using pulldown, co-immunoprecipitation, isothermal titration calorimetry, and FRET experiments, Bernlohr and co-workers (37, 54) demonstrated that A-Fabp interacts with Hsl. In contrast to the A-Fabp/Cgi-58 interaction, Hsl only interacts with A- and E-Fabp but not with L- or I-Fabp, and the A-Fabp/Hsl interaction only occurs when A-Fabp is loaded with a FA (36, 55). The contact region between A-Fabp and Hsl involves a cluster of four residues (Asp-18, Asp-19, Lys-22, and Arg-31) (37) and the authors hypothesized that this region may be a general motif for protein/protein interactions of A-Fabp. However, solid phase assays with mutant A-Fabp variants revealed that Asp-18, Lys-22, and Arg-31 were not required for binding of A-Fabp to Cgi-58. These data suggest that Hsl and Cgi-58 do not share an identical contact region.

The interaction of A-Fabp and Cgi-58 has important functional consequences. First, A-Fabp promotes Atgl-mediated TG hydrolysis. Consistent with our findings that A-Fabp only binds to Cgi-58 but not to Atgl, the activation of Atgl's hydrolytic activity by A-Fabp depends on the presence of Cgi-58. Apparently, Cgi-58 acts as an adaptor protein enabling A-Fabp to bind the FA produced by Atgl.

Inhibition of A-Fabp by the specific inhibitor BMS309403 led to markedly reduced TG hydrolase activities in WAT and 3T3-L1 cell lysates. Inhibition of TG hydrolase activity by BMS309403 was observed in the presence of an Hsl inhibitor but not in the presence of an Atgl inhibitor providing compelling evidence that A-Fabp stimulates Atgl/Cgi-58-mediated lipolysis. In our view, direct binding of A-Fabp to the Atgl/Cgi-58 complex or Hsl is required to sequester FAs from the first and the second step of TG hydrolysis. The immediate FA capture *in statu nascendi* likely prevents product inhibition by increased FA concentrations as both Hsl (56) and Atgl (57) are known to be inhibited by high concentrations of oleic acid. In accordance with this conclusion, genetic abrogation of A-Fabp in mice leads to reduced lipolysis (58).

Besides their crucial role as energy substrates and membrane lipid precursors, FAs also act as important signaling molecules. Among many other ligands, they can bind to members of the Ppar family of nuclear receptors that dimerize with retinoid X receptor to regulate the expression of numerous genes involved in energy metabolism (59). The nuclear import of FAs has been shown to involve L-Fabps to activate Pparα in hepatocytes, K-Fabp to activate Pparβ/δ, or A-Fabp to activate Pparγ in adipocytes. Fabps bind to Ppars in the nucleus, and it is assumed that they “hand over” the FA to the Ppar ligand-binding site (40, 43). Nuclear translocation of Fabps appears to depend on the stabilization of an “activated” state of Fabps by a subset of small molecule ligands (60–63).

We show that the transfection of COS-7 cells with either Pparα or Pparγ and a luciferase reporter construct under the control of multiple PPREs leads to increased luciferase reporter gene expression when lipolysis is increased by co-expression of Atgl and Cgi-58. Lipolysis-induced transcription increased further when COS-7 cells were additionally transfected with L-Fabp- or A-Fabp-expressing plasmids. Mutant A-Fabp variants that either cannot bind FAs or exhibit impaired nuclear translocation were unable to stimulate Pparγ-activated reporter gene expression. These data support the concept that the provision of specific FAs by lipolysis, FA binding by Fabps, and the nuclear translocation of Fabps are all important for Ppar signaling. The interaction between Cgi-58 and A-Fabp may play a crucial role in this process. Interestingly, residue Phe-58, which together with the nuclear localization signal residues Lys-22, Arg-31, and Lys-32, which mediates the nuclear import of A-Fabp (62), is also part of the A-Fabp/Cgi-58 binding interface. Because the interaction of both proteins does not prevent A-Fabp nuclear translocation, we speculate that the binding of A-Fabp to Cgi-58 occurs transiently on the surface of LDs, where FAs are generated by the Atgl reaction. It is also conceivable that the quick FA binding to Fabps prevents their activation by acyl-CoA synthetases and permits their translocation to the nucleus in an unactivated state.

Another important aspect of the A-Fabp/Cgi-58 interaction refers to a FA transport-independent function of A-Fabp. Similar to other regulatory factors involved in lipolysis (*e.g.* Cgi-58, Pedf, or G0s2), A-Fabp also has demonstrated bioactivities unrelated to its function in FA transport and lipolysis. For example, A-Fabp has been shown to act as an adipokine regulating hepatic glucose production (64) or induces the ubiquitination and degradation of Pparγ (65). Whether and how these activities of A-Fabp are affected by its interaction with Cgi-58 remains elusive and requires clarification.

In summary, we show that Fabps interact with the Atgl co-activator Cgi-58. This interaction represents the basis for a novel mechanism to modulate lipolysis and Ppar-mediated gene expression.

## Footnotes

3 The abbreviations used are:

TG: triglyceride
Atgl: adipose triglyceride lipase
Cgi-58: comparative gene identification-58
FA: fatty acid
Fabp: fatty acid-binding protein
Hsl: hormone-sensitive lipase
LD: lipid droplet
Pedf: pigment epithelium derived factor
Ppar: peroxisome proliferator-activated receptor
PPRE: peroxisome proliferator-activated receptor responsive element
WAT: white adipose tissue
E-Fabp: epidermal-Fabp
L-Fabp: liver-Fabp
I-Fabp: ileum-Fabp
H-Fabp: heart-Fabp
TEV: tobacco etch virus
HSQC: heteronuclear single quantum coherence spectroscopy.
